# Synchrotron CT of an equine digit at the Australian Synchrotron Imaging and Medical Beamline

**DOI:** 10.1107/S1600577521010493

**Published:** 2021-10-22

**Authors:** J. B. Montgomery, M. Klein, J. R. Boire, C. Beck, D. Häusermann, A. Maksimenko, C. J. Hall

**Affiliations:** a Department of Large Animal Clinical Sciences, University of Saskatchewan, Saskatoon, SK S7N 5B4, Canada; b Division of Biomedical Engineering, University of Saskatchewan, Saskatoon, SK S7N 5B4, Canada; c Imaging and Medical Beamline, Australian Synchrotron (ANSTO), Wurundjeri Country, Clayton, VIC 3168, Australia; d RMD Engineering Inc., Saskatoon, SK S7K 3J7, Canada; e University of Melbourne, Werribee, VIC 3030, Australia

**Keywords:** horses, laminitis, laminar blood flow, laminar imaging

## Abstract

Laminitis is an extremely painful and debilitating condition of horses that can affect their athletic ability and even quality of life. The current gold standard for assessment of laminar tissue is histology, which is the only modality that enables detailed visualization of the lamina. Histology requires dissection of the hoof and therefore can only represent one specific time point. The superior spatial and contrast resolution of synchrotron computed tomography (sCT), when compared with readily available imaging modalities, such as radiographs and conventional CT, provides an opportunity for detailed studies of the lamina without the need for hoof dissection and histological assessment. If the resolution of histology can be matched or even approached, dynamic events, such as laminar blood flow, could also be studied on the microscopic tissue level. To investigate this possible application of sCT further, two objectives are presented: (i) to develop a protocol for sCT of an equine digit using cadaver limbs and (ii) to apply the imaging protocol established during (i) for sCT imaging of the vasculature within the foot using an *ex vivo* perfusion system to deliver the vascular contrast. The hypotheses were that sCT would allow sufficient resolution for detailed visualization to the level of the secondary lamellae and associated capillaries within the equine digit. Synchrotron CT enabled good visualization of the primary lamellae (average length 3.6 mm) and the *ex vivo* perfusion system was able to deliver vascular contrast agent to the vessels of the lamina. The individual secondary lamellae (average length 0.142 mm) could not be seen in detail, although differentiation between primary and secondary lamellae was achieved. This approaches, but does not yet reach, the current gold standard, histology, for assessment of the lamellae; however, with further refinement of this imaging technique, improved resolution may be accomplished in future studies.

## Abstract 2

The applicability of synchrotron computed tomography (sCT) for the study of equine laminar structures is investigated, as well as its potential to study laminar blood flow. Based on the findings, sCT imaging has the potential to be an important addition to the ‘tool kit’ currently used by researchers aiming to better understand the many factors that can lead to the development of equine laminitis.

## 1. Introduction and objectives

‘No foot, no horse’ – nothing highlights better the fate suffered by horses that lose structural integrity of their feet. In the horse, each foot (digit) is reduced to one toe, the distal phalanx or pedal bone, and the horse’s entire weight (500 kg in an average-sized horse) rests on each of the four distal phalanges suspended within a solid hoof capsule. The suspension, also known as suspensory apparatus of the distal phalanx (SADP), is enabled by the lamina, which connects the hoof bone to the hoof capsule like Velcro (Pollitt, 2010 ▸). Disruption of this connection, laminitis, can result in failure of the suspension and subsequent displacement (rotation and sinking) of the hoof bone within the hoof capsule. Such failure is at best difficult and at worst impossible to reverse, frequently ending the horses’ career and even necessitating euthanasia for humane reasons in severe cases. Both its severity and frequency make laminitis a serious welfare issue (Pollard *et al.*, 2019 ▸) and are the reason why investigations into laminitis pathophysiology, treatment and prevention continue to be a high priority of researchers, equine veterinarians and the entire equine industry (Katz & Bailey, 2012 ▸; The Horse, 2019 ▸).

Laminitis can be a consequence of sepsis, endocrine dysfunction, or mechanical overload with proposed alterations in digital blood flow contributing to its development. These different clinical forms probably share some pathophysiological mechanisms (van Eps & Burns, 2019 ▸; Patterson-Kane *et al.*, 2018 ▸). Both acute and chronic manifestations of laminitis are observed, affecting one or more feet at a time. The lamina, which facilitates the connection between the distal phalanx and hoof capsule, is modified skin tissue with an epidermal and dermal component (Parks, 2017 ▸). Histologically, it is made up of interdigitating epidermal and dermal primary lamellae (average length 3.6 mm), each of which are made up of secondary lamellae (average length 0.142 mm) to increase surface area (Pollitt, 2017 ▸).

One of the challenges associated with studying laminitis is the anatomical enclosure of the distal phalanx and associated structures within the hoof capsule, preventing direct assessment in live animals; there are increasing ethical concerns about experimental induction of this extremely painful condition. Because of the enclosure within the hoof capsule, tissue samples from the lamina can only be collected and examined post mortem, and therefore only reflect one time point in the disease stage. Horses with experimentally induced acute laminitis cannot be kept alive very long after disease onset for humane reasons, limiting the types of observations in such studies (van Eps & Pollitt, 2006 ▸). The gold standard in post-mortem examination is micro-histological examination.

Non-invasive evaluation of the equine digit and its surrounding soft tissue structures inside the hoof capsule can be achieved with different medical-imaging modalities. Traditional radiographs, computed tomography (CT) and, more recently, magnetic resonance imaging (MRI) are used to image the equine hoof (Pownder *et al.*, 2020 ▸). Even though MRI allows for visualization of soft tissue structures such as ligaments within the hoof capsule, it cannot achieve the resolution required to study the primary and secondary lamellae in detail; such observation is currently limited to dissection of the hoof and subsequent histological assessment.

Blood flow within the equine digit is facilitated by loading and unloading of the hoof, with the hoof functioning similar to a peristaltic pump. Blood can either flow through a capillary bed or shunt directly from the arterial to the venous vascular system through arterio-venous anastomoses (Peroni *et al.*, 2017 ▸). The laminar capillaries are located only in the secondary dermal lamellae (Pollitt, 2016 ▸). Changes in microvascular blood flow are thought to contribute to laminitis development (van Eps & Burns, 2019 ▸; Medina-Torres *et al.*, 2016 ▸), and venograms are used to monitor laminitis recovery and aid in establishing a prognosis for affected horses (Baldwin & Pollitt, 2010 ▸). In a research setting, post-mortem arterial contrast studies have been performed to aid in the understanding of digital blood flow in laminitis associated with excessive and prolonged weight bearing (van Eps *et al.*, 2010 ▸). This type of laminitis is also known as supporting-limb laminitis (SLL). Based on van Eps *et al.* (2010 ▸), moderated loading decreased the arterial contrast delivered to the foot and heavy loading resulted in complete absence of arterial-contrast fill below the coronary band. These findings led to the conclusion that load-associated ischemia and lack of glucose delivery probably play a role in the development of SLL. One study indirectly assessed changes in laminar blood flow in response to limb loading through the measurement of metabolites as they were delivered to and returned from the foot (Medina-Torres *et al.*, 2016 ▸); however, this approach does not enable differentiation between the proportion of blood flow directed though capillaries versus arterio-venous anastomoses. Despite these research efforts, the anatomical and experimental challenges associated with studying the lamina directly *in situ* mean that detailed knowledge of microvascular blood-flow alterations during different types and stages of laminitis is still lacking.

Since both mechanical overload and load-induced ischemia can contribute to breakdown of the SADP, members of our research team (J. R. Boire and J. B. Montgomery) have worked on development and testing of a computer-controlled weight-reduction system to aid in the rehabilitation of horses (Montgomery *et al.*, 2019 ▸). In order to provide more data to set the operating parameters for the cyclic loading on the weight reduction system, we need to gain a better understanding of the effect of incremental loading on laminar capillary blood flow.

The CT at the Australian Synchrotron (ANSTO) Imaging and Medical Beamline (IMBL) has the potential to provide enough resolution (Stevenson *et al.*, 2012 ▸) for direct detailed assessment of the lamina without the need for hoof dissection and histological assessment. Achieving resolution to the level of the secondary lamellae without dissection of the hoof would greatly contribute to the research effort of understanding underlying pathophysiological processes in the different types and stages of laminitis, therefore advancing laminitis research and ultimately the welfare of horses.

To investigate this possible application of synchrotron CT (sCT) further, we had two objectives. Objective (i) to develop a protocol for synchrotron-based CT of an equine digit using cadaver limbs. Specific aims were to: determine the optimal imaging technique, optimum resolution and phase contrast to visualize laminar structures in normal and laminitic horse feet, to assess if freezing of the cadaver limbs prior to imaging affects image quality, and to determine how closely the acquired images will resemble the current gold standard – histology. Objective (ii) to apply the imaging protocol established during (i) for sCT imaging of the vasculature within the foot using an *ex vivo* perfusion system to deliver the vascular contrast agent. Our hypotheses were that sCT would allow sufficient resolution for detailed visualization to the level of the secondary lamellae and associated capillaries within the equine digit.

## 2. Methods

### 2.1. Ethics statement

Horse cadaver limbs were obtained from a local abattoir and a local equine veterinary clinic. None of the animals were euthanized for the purpose of the study.

### 2.2. Beamline setup

**Figure 1:**
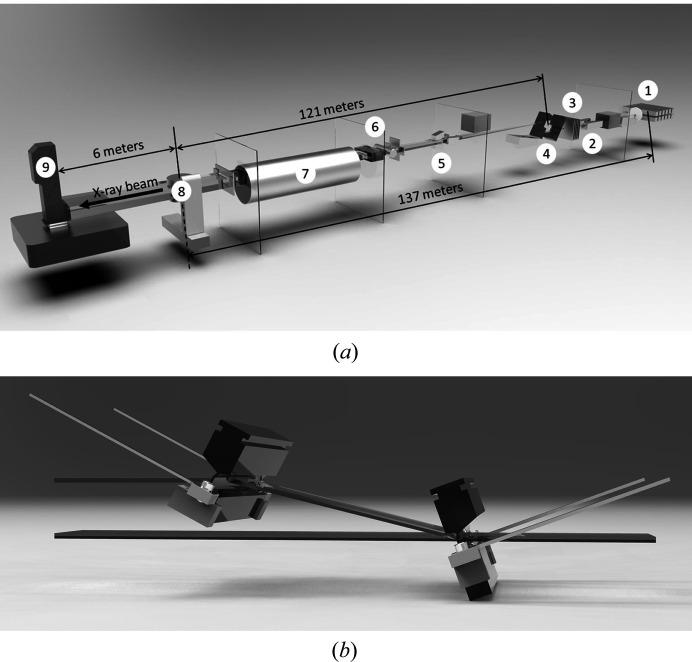
Layout of the IMBL (*a*) with (1) the X-ray source, a 62 poles, 4.2 Tesla superconducting wigger (Häusermann *et al.*, 2010 ▸); (2) beam-defining slits; (3) filters to remove low-energy X-rays and reduce the thermal load on the monochromator; (4) a double-crystal Laue-type monochromator; (5) secondary slits; (6) clean-up slits, an imaging shutter and an ionization chamber to monitor the beam intensity; (7) a 90 m-long beam-transfer line to the IMBL satellite building with enclosure 3B (see the main text); (8) pre-sample clean-up slits; (9) a multi-axes CT stage; and (10) the Ruby imaging detector. The IMBL is 145 m long with a maximum sample-to-detector distance of 8 m. (*b*) The monochromator used to acquire sCT images at the Australian Synchrotron IMBL.

A schematic diagram of the IMBL is shown in Fig. 1 ▸(*a*). For this work, the wiggler field was set at 3 Tesla with a filtration of 22 mm of graphite and 2.8 mm of aluminium. The X-ray detector used in this first part of our work was an indirect scintillator-optics-camera device named ‘Ruby’. Ruby uses a 25 µm-thick Gadox P43 (Gd2O2S: Tb) screen to convert the incident X-ray field to optical light (Hall *et al.*, 2013 ▸). The screen is viewed through a silver-coated mirror set at 45°, by a scientific CMOS sensor. This design is moderately efficient but avoids radiation damage to the sensitive sensor electronics. Ruby uses a commercial macro lens as a light collector, with a variable focal distance allowing image pixel sizes to be set between 6 and 25 µm. For our experiment, the pixel size was 12.0 µm with a corresponding detector field of view of 30.7 mm (H) × 25.9 mm (V). To completely image the width of the specimens, projection images were collected over two rotations of 360° with their centres separated by a horizontal offset of 25 mm. To cover the vertical region of interest, four CT slices were collected with the specimen moved vertically between each.

All scans in this work included background (beam without the sample) and dark field (detector noise without the beam) imaging before and after the sample image sets. Each sample projection was then corrected using the medians of 100 background and 100 dark-field images.

At the start and end of each projection-image set, a series of calibration images were captured. One hundred flat-field and dark-field images were taken, with no sample in the beam and with the beam turned off, respectively. An average of these images was used to calibrate systematic pixel-to-pixel differences in illumination and efficiency. After calibration, the projection-image pixel values are the true ray transmission through the specimen.

The IMBL allows for simple tuning of the beam energy using a double-crystal Laue-type monochromator [Fig. 1 ▸(*b*); Stevenson *et al.*, 2017 ▸]. For this experiment, we used energies between 55 and 80 keV, favouring 55 keV for most data sets. This energy balances the competing demands for spatial resolution, penetration and propagation distance.

To exploit the X-ray beam coherence for phase-contrast imaging, the detector and sample were separated by 6.0 m. The effect of this is to allow X-rays refracted at the boundaries of tissue densities to interfere during propagation before impinging on the detector. This results in a raw image with a slight edge enhancement. By measuring these interference fringes, we can use the homogeneous transport of intensity equation (TIE-Hom) to calculate phase shifts of the X-rays through the specimen (Paganin *et al.*, 2002 ▸). This increases the contrast resolution in the projection images at the cost of a slight reduction in spatial resolution. TIE-Hom makes an assumption of a single material in the specimen. Although not realistic in this case, the approximation yields a valuable phase-contrast benefit and greatly simplifies the image-collection protocol.

Initial processing of the raw projection-image data was with the in-house *IMBL-Stitch* software (Maksimenko, 2018 ▸). Standard flat- and dark-field corrections were applied, and the vertical images were stitched. Reconstruction and phase retrieval were subsequently carried out using the CSIRO *XTRACT* program (Gureyev *et al.*, 2011 ▸). Here the TIE-Hom phase-retrieval algorithm was applied before using filtered back projection (FBP) to reconstruct the 3D volume. A key parameter used in the TIE-Hom algorithm is the ratio between the real and imaginary parts of the pseudo-material refractive index. In our processing, a value of 100 was found to be optimal. Output was in the form of a stack of TIFF images. The image format was real 32-bit floating point. Each voxel value being the calculated X-ray attenuation coefficient at this beam energy.

### 2.3. Description and preparation of cadaver limbs

Nine equine cadaver limbs were obtained from a local abattoir on the day the limbs were perfused (fresh). Additionally, four previously frozen equine limbs were included in the study (frozen). Two of the frozen limbs were obtained from a horse that was euthanized because of severe acute laminitis; these were provided by a local equine veterinary clinic.

The fresh limbs were prepped for infusion of vascular contrast agent with an *ex vivo* perfusion system, as described in the following section (day one). A 14-gauge Mila catheter was inserted into both the lateral and medial digital artery at the level of the fetlock (metacarpo- or metatarso-phalangeal) joint of each fresh cadaver limb and secured with #2 Daclon (nylon) suture. Prior to perfusion and after perfusion, until CT and sCT imaging, the fresh limbs were stored in a fridge at +4°C. The frozen limbs were stored in a freezer at −20°C. Information on the preparation of each cadaver limb, as well as the data obtained from each limb, are summarized in Table 1 ▸.

### 2.4. *Ex vivo* vascular perfusion system for delivery of vascular contrast agent

The *ex vivo* perfusion system was built by RMD Engineering Inc. (Saskatoon, Saskatchewan, Canada) to deliver the vascular contrast agent while cyclically loading and unloading the limb. It is a modified version of the system previously described (Patan *et al.*, 2009 ▸). The RMD Engineering manufactured cantilever press has a 10:1 lever arm, with the pivot of the lever being 60 mm from the vertical swivel mounting hole. The force arm length is ∼600 mm long, and a scale was attached to the end of the lever arm to indicate force applied to the limb and hoof. A Model 45MPHP2 peristaltic pump was used to deliver the perfusate and the discharge flowed through a Hydor Model ETH 300 vertical fluid heater and pressure control valve. The pressure was monitored by a standard 0 to 3 psi (0 and 155 mmHg) pressure gauge. The output was attached to a catheter inserted into the digital artery, as described above. The pump was turned on at a fixed 30 r min^−1^ and pressure was monitored between 5 and 80 mmHg, while the perfusate was delivered at a rate of 25 ml min^−1^ until 500 ml was perfused through the digit. During the perfusion, 245 N was cycled onto the lever arm resulting in a resultant force on the limb of ∼2.45 kN and returned to 0 at a rate of ∼30 times a minute.

**Figure 2:**
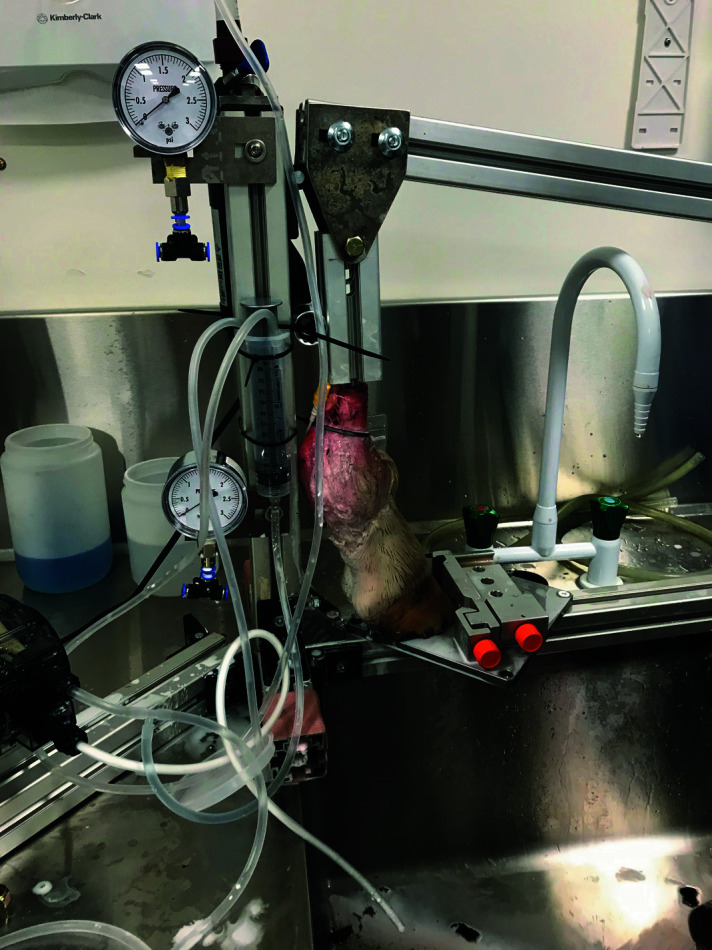
An equine cadaver foot inserted into a cantilever press to facilitate cyclic loading and unloading during *ex vivo* perfusion with vascular contrast.

The fresh cadaver limbs were trimmed from the bottom of the flat hoof to the top of the excised limb to an overall length of ∼280 mm. The centre of the bone was then drilled out and threaded as perpendicular to the bone as possible, to accept an M12 × 1.75 mm × 60 mm stud. The stud was inserted into the limb to ∼30 mm in depth, and then the stud was threaded into the vertical swivel. The vertical swivel was inserted into the cantilever arm and the swivel retention bolt was then inserted to retain the assembly in the cantilever press (Fig. 2 ▸).

Prior to infusion of contrast agent, the vasculature of each equine cadaver foot was flushed with 500 ml of heparinized 0.9% NaCl (5000 IU heparin litre^−1^). The vascular contrast agent used was BriteVu (Scarlet Imaging, Utah, USA), which is a commercial barium-based compound specifically intended for post-mortem vascular imaging (Clark & Badea, 2021 ▸; Xie *et al.*, 2019 ▸). One cadaver limb was infused with commercial iodixanol (Visipaque, GE Healthcare, New South Wales, Australia), a commonly used contrast agent in veterinary imaging, for comparison. Eight of the nine fresh limbs were perfused with contrast agent (seven with BriteVu, one with Visipaque), one limb remained unperfused to enable imaging of a fresh unperfused limb for comparison. Contrast agent was prepared according to the manufacturer’s instructions and 500 ml was infused via the digital arteries of each limb.

### 2.5. Conventional CT

Conventional CT (cCT) was performed with a 32-slice Siemens Somatom GoUp clinical CT machine located at the Monash Biomedical Imaging research platform, next to the IMBL, on day three. All 13 cadaver feet (nine fresh, four frozen) were imaged. The purpose was to compare the resolution of the cCT with that of the sCT and to assess the distribution of vascular contrast agent in the fresh feet since the feet were perfused post mortem. The slice collimation was 0.6 mm with overlap and images were reconstructed at 0.3 mm resolution. The X-ray source was 80 kVp, with a tube current of 95 mA. Scan duration was 120 s. Scans were reconstructed with the HR60 S3 kernel settings. Images were collected in DICOM files. Results were used to help determine which feet should undergo sCT imaging in order to make more efficient use of the available IMBL beam time.

### 2.6. Synchrotron CT

**Figure 3:**
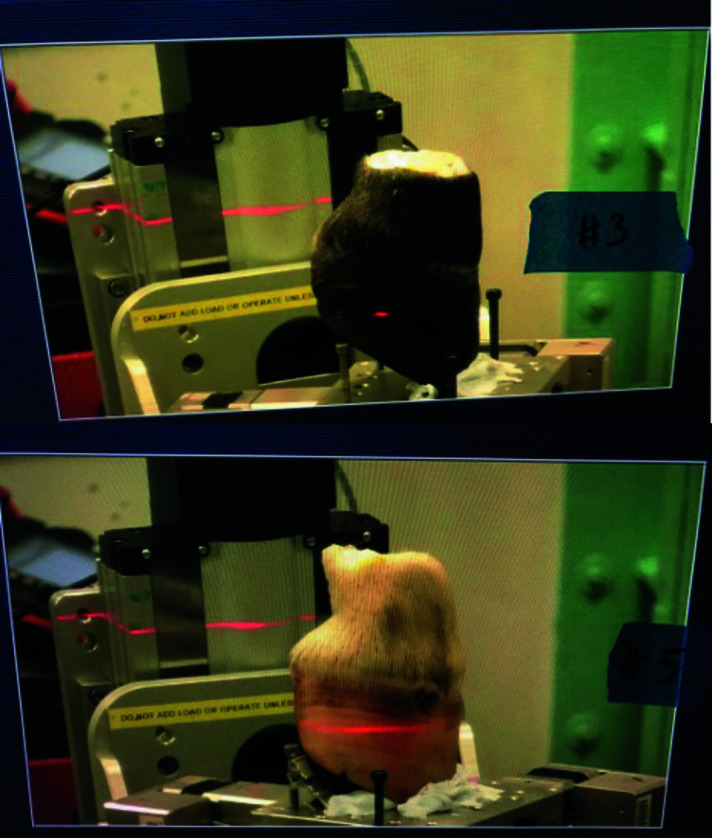
Positioning of equine cadaver feet #3 and #5 during sCT imaging at the Australian Synchrotron IMBL.

These comparative sCT scans were performed on the IMBL on days four and five. Prior to imaging of each cadaver foot, to facilitate positioning within the beam, each of the fresh limbs was sawn off just above the pastern (proximal interphalangeal) joint. Positioning within the beam was the same for all cadaver feet and is shown in Fig. 3 ▸.

Synchrotron CT scans were conducted in Hutch 3B of the IMBL (Häusermann *et al.*, 2010 ▸). These scans were also performed with each hoof at 6 m from the detector as the long distance increases the propagation phase contrast. For this part of the work the detector was changed to a Hamamatsu C9252-DK14 flat panel device with 100 µm pixels and a field of view of 126 mm to cover the hooves width in a single image. We collected 3600 images over 360° at 0.059 second per image.

The sCT data sets were collected with the hoof displaced from the centre of rotation, then flipped and stitched in the pre-processing to obtain one full image every 0.1°. The scans included background and dark-field images imaging before and after the sample, image sets, and corrections of the latter using the median of 100 frames, as described earlier. Images were collected in TIF files and viewed on *Fiji* (Abràmoff *et al.*, 2004 ▸) software. They were converted to JPG files for this publication. All feet were frozen at −80°C after completion of the scan. Feet used for the long scans (hours) were disposed of.

#### 2.6.1. Objective (i) – to develop a protocol for synchrotron-based CT of the equine digit using cadaver limbs

*Optimal energy (keV) and optimal distance from the detector.* Hoof #9 (fresh, no contrast) was imaged repeatedly to determine optimal keV and detector distance (m) for high-resolution imaging of the equine lamina. The different scan settings that were assessed are listed in Table 2 ▸. The foot was kept in the same position and the same slice was imaged with each setting for best possible comparison. A full rotation (short scan) of hoof #9 was imaged once the final settings to be used for optimum resolution had been determined.

*Comparison of fresh and frozen cadaver limbs.* Hoof #10 (frozen) was imaged with the established short-scan protocol to determine if prior freezing affects image quality.

*Imaging of cadaver feet from a horse with laminitis.* Hooves #12 and #13, the right and left front feet from a horse with laminitis, were imaged with the same short-scan protocol as hooves #9 and #10. Hoof #13 also underwent a long scan, imaging the entire hoof from the sole to the coronary band.

#### 2.6.2. Objective (ii) – to apply the imaging protocol established during Objective (i) for sCT imaging of the vasculature within the foot using an *ex vivo* perfusion system to deliver the vascular contrast agent

Hooves #1 (Visipaque), #3 and #5 (BriteVu) were imaged with the short-scan protocol established in Objective (i). Hoof #5 also underwent a long scan, similar to hoof #13.

### 2.7. Histology

Histologic assessment of four horse feet, previously frozen at −80°C, was completed. Hoof sectioning was performed by the Veterinary Pathology Department at the University of Melbourne as previously described to enable histological assessment of the lamina (Pollitt, 1996 ▸). Hooves sectioned for histology included normal with no contrast, normal with contrast and hoof with laminitis (Table 1 ▸). These were not the same feet that were used during the synchrotron-based CT imaging to rule out radiation-induced tissue changes.

## 3. Results and discussion

**Figure 4:**
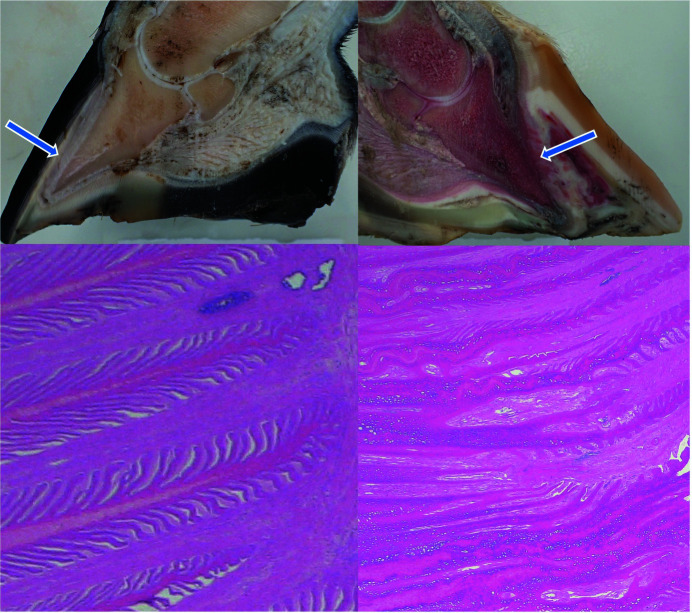
Cross section through a normal hoof (top left) and a hoof from a horse with acute laminitis (top right). The arrows indicate the dorsal hoof wall covered by dermal lamina. The pedal bone in the normal hoof is parallel to the hoof wall whereas it is rotated away from the dorsal hoof wall in the hoof obtained from the horse with laminitis. Bottom left and bottom right images show a hematoxylin and eosin stain of a histology slide from a normal horse and the horse with laminitis, respectively (magnification 1× objective). The top right hoof is the right front hoof (#12) also depicted in the radiographs obtained from the horse with acute laminitis prior to euthanasia (Figs. 9 ▸ and 10 ▸).

Fig. 4 ▸ shows the normal position of the distal phalanx within the hoof capsule (cadaver-limb cross section) and an example of structural changes associated with rotation of the hoof bone as a consequence of laminitis. The figure further provides an example of the histological appearance of normal primary lamellae and those from a horse suffering from severe acute laminitis.

### 3.1. Conventional CT

**Figure 5:**
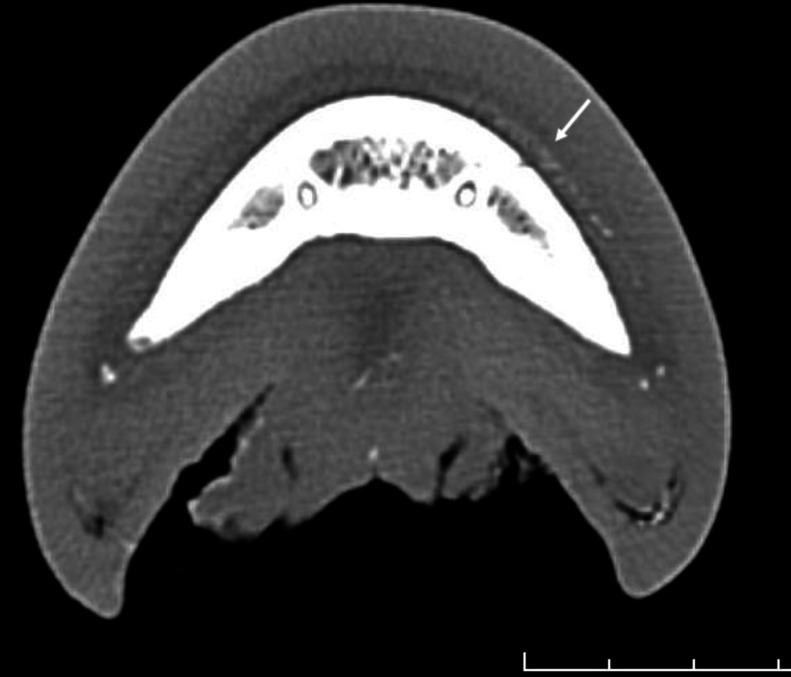
Hoof #5 taken with cCT. The BriteVu contrast is seen within the lamellar capillaries (arrow). The filling of the capillaries is moderately asymmetrical.

Hooves #3 and #5 had the best distribution of BriteVu contrast and were chosen for the sCT scans. Overall, BriteVu provided better post-mortem vascular contrast than Visipaque. With the help of the perfusion system, BriteVu contrast material could be delivered to the level of the lamellar capillaries (Fig. 5 ▸). The filling of the capillaries was moderately asymmetrical.

### 3.2. Synchrotron CT

Specimen characteristics and images acquired from each equine cadaver foot are summarized in Table 3 ▸. Seven of the 13 cadaver feet underwent sCT imaging. The number was limited by the duration of each scan and the available overall beam time (two days). The area of interest was the lamina, located between the hoof bone and the hoof capsule. Initial imaging of hoof #9 (Table 2 ▸) showed that an energy of 55 keV and a specimen distance from the detector of 6 m resulted in optimal resolution of the lamina. These parameters were therefore used for all subsequent sCT scans. Short scans represent one section (one full rotation) of the hoof. All seven feet underwent a short scan obtained with the same positioning of the foot on the stage and within the beam (Fig. 3 ▸). Imaging of the entire hoof from the sole to the coronary band was performed on hoof #5 (fresh, BriteVu) and hoof #13 (frozen, laminitis).

**Figure 6:**
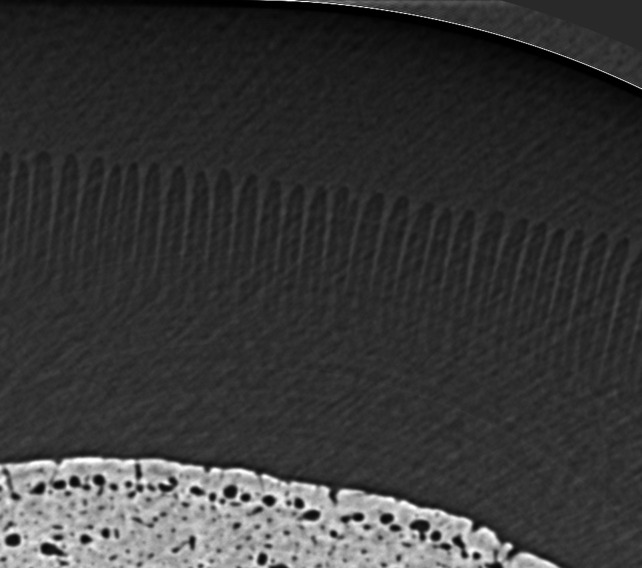
Hoof #9 of a normal horse with no contrast. The primary lamellae are the finger-like projections extending into the hoof capsule. The individual secondary lamellae are not viewable; however, the dark band on the external surface of each primary lamellar represents the secondary lamellae.

**Figure 7:**
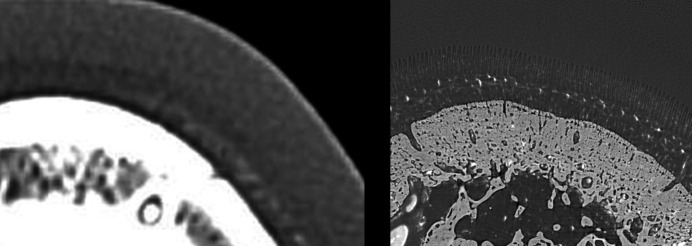
Hoof #5 of a normal horse with BriteVu contrast. Comparison of cCT and images obtained on the synchrotron. The spatial superior resolution of the sCT enables the visualization of the laminae.

Fig. 6 ▸ shows an image acquired during sCT imaging of hoof #9. The individual secondary lamellae cannot be seen however; the dark band on the external surface of each primary lamellar represents the secondary lamellae. When comparing conventional and synchrotron CT the superior spatial resolution of the sCT enables the visualization of the laminae (Fig. 7 ▸).

**Figure 8:**
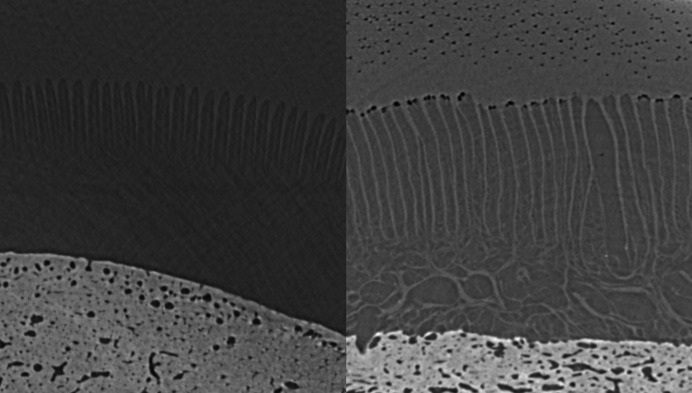
Comparison of the images obtained from hoof #9, fresh specimen, (left) and hoof #10, frozen specimen (right), with no contrast. There is moderate distortion and elongation of the lamellae on hoof #10 but they remain moderately parallel.

**Figure 9:**
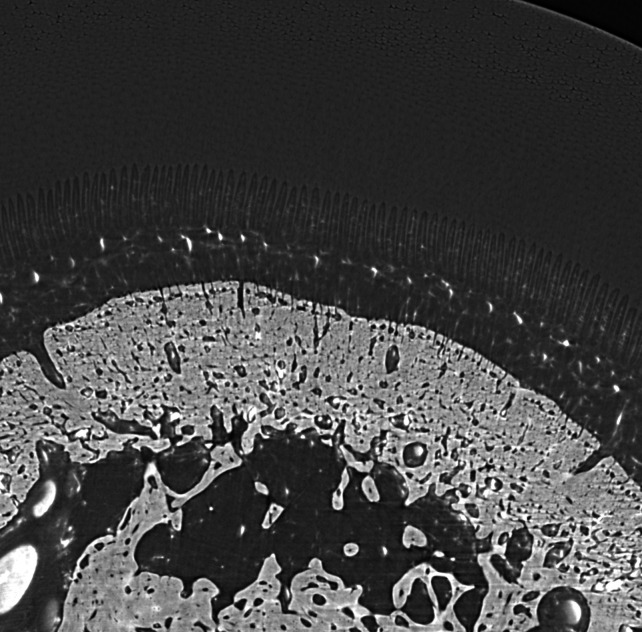
Hoof #5 of a normal horse. The BriteVu contrast is distributed within the vessels of the lamellae.

A comparison of the image quality when imaging fresh versus frozen hooves (hoof #9 versus hoof #10) is shown in Fig. 8 ▸. There is moderate distortion and elongation of the lamellae on hoof #10; however, they remain moderately parallel. Fig. 9 ▸ shows the distribution of BriteVu contrast within the vessels of the lamellae.

### 3.3. Imaging of cadaver feet from a horse with laminitis

#### 3.3.1. Radiographs

**Figure 10:**
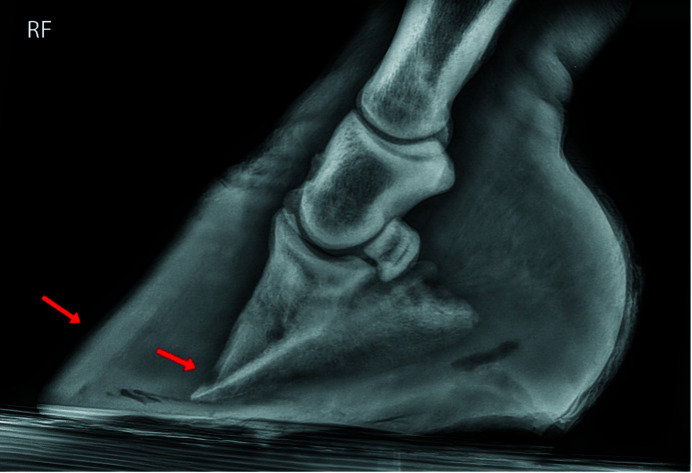
A radiograph of the right front foot (RF, hoof #12) obtained from a horse with severe laminitis, prior to euthanasia, with evidence of rotation (red arrows) of the pedal bone within the hoof capsule. Radiographs were taken by Ballarat Equine Clinic and are provided here with their permission.

**Figure 12:**
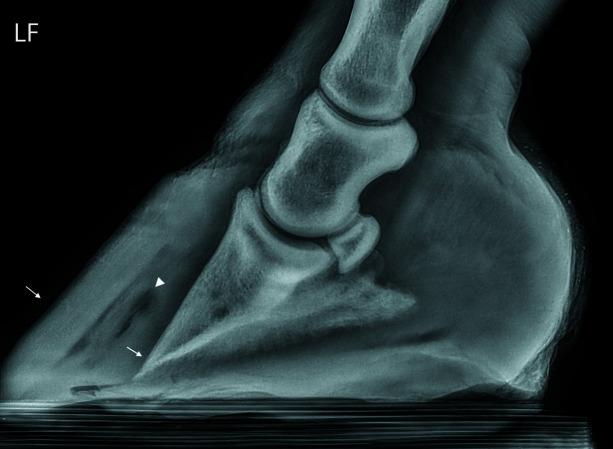
A lateromedial radiograph of the left front foot (LF, hoof #13) of the horse with laminitis. The distal phalanx has rotated and is no longer sitting parallel to the dorsal hoof wall (arrows). There is an accumulation of serum within the hoof wall (arrowhead), a characteristic finding in horses with laminitis.

Lateral radiographs of both the right (hoof #12) and left (hoof #13) front foot were taken of the horse, a five-year-old Gypsy Cob mare, at Ballarat Equine Clinic prior to euthanasia because of severe laminitis that was not responding to treatment. The suspected cause of the laminitis was endocrine (hyperinsulinemia-associated laminitis as a consequence of insulin dysregulation seen with Equine Metabolic Syndrome); a definitive diagnosis was not provided. Radiographs of both front feet show severe structural changes that can occur in horses with laminitis, including evidence of rotation of the hoof bone away from the dorsal hoof wall and sinking of the hoof bone towards the sole, with more severe changes in the right front foot (Figs. 10 ▸ and 11 ▸). Fig. 12 ▸ further shows accumulation of serum within the hoof wall (arrowhead), a characteristic finding in horses with laminitis. A cross section of hoof #12 as well as an example of the histological changes of the lamina obtained from the same hoof can be seen in Fig. 4 ▸. Characteristic histological changes in laminitis include elongation and thinning of the lamellae.

#### 3.3.2. Conventional and synchrotron CT

**Figure 13:**
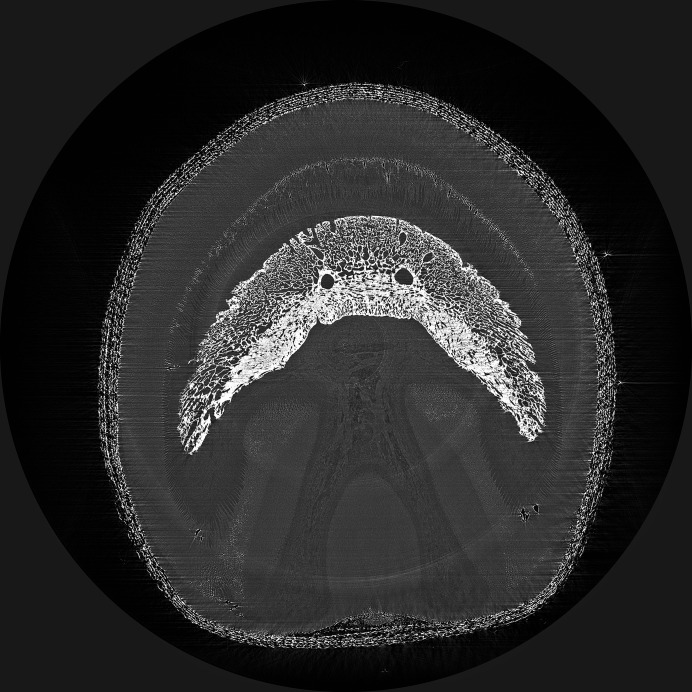
Hoof #13 of the laminitis horse, full view with no contrast. This hoof (left front) is from the same horse as in Figs. 11 ▸ and 12 ▸. The lamellae are no longer parallel. Some of the lamellae are shortened and others are thickened and distorted.

Conventional CT did not provide enough resolution for assessment of the lamina. In the sCT of one of the hooves affected by laminitis (Figs. 13 ▸ and 14 ▸) the lamellae are no longer parallel. Some of the lamellae are shorted and others are thickened and distorted. The darker areas may represent areas of serum accumulation. These changes are consistent with histological findings in horses with laminitis.

## 4. Conclusions

This is the first work investigating the use of sCT for detailed study of equine lamina and its potential for phase-contrast imaging of laminar blood flow. The first objective was to develop a protocol for sCT of an equine digit using cadaver limbs. In this study performed at the IMBL of the Australian Synchrotron (ANSTO), we found that an energy of 55 keV and a specimen distance from the detector of 6 m resulted in optimal resolution of the lamina. Intravascular contrast material specifically formulated for post-mortem studies was able to diffuse into the small laminar vessels with the help of a specifically designed cadaver-limb perfusion system. Freezing appeared to result in only minor structural alterations. Synchrotron CT enabled good visualization of the primary lamellae (average length 3.6 mm). The individual secondary lamellae (average length 0.142 mm) could not be seen in detail but a dark band on the external surface of each primary lamellar represents the secondary lamellae. This approaches, but does not yet reach, the current gold standard for assessment of the lamellae, histology; however, with further optimization of the imaging technique, improved resolution may be accomplished in future studies. Another option may be to use spectral *k*-edge subtraction imaging (Zhu *et al.*, 2014 ▸) around the imaging energy of barium to verify that the loading of the hoof during perfusion will drive the contrast agent down to the secondary lamellae; however, the fact that the secondary lamellae have a different colour in the images obtained in this study (Figs. 8 ▸ and 9 ▸) indicates differentiation between the two tissues, so better resolution may not be necessary for the study of capillary blood flow.

The initial testing of an *ex vivo* perfusion system to deliver vascular contrast agent for sCT imaging of the vasculature within the foot and specifically the lamina yielded promising results. These results will enable us to use the *ex vivo* perfusion system tested here to visualize the effect of loading and unloading, as well as different loads, on laminar blood flow. These studies will contribute to developing our understanding of how loading affects laminar blood flow with the ultimate goal of studying its role in laminitis development and prevention in live horse imaging.

## Figures and Tables

**Figure 11:**
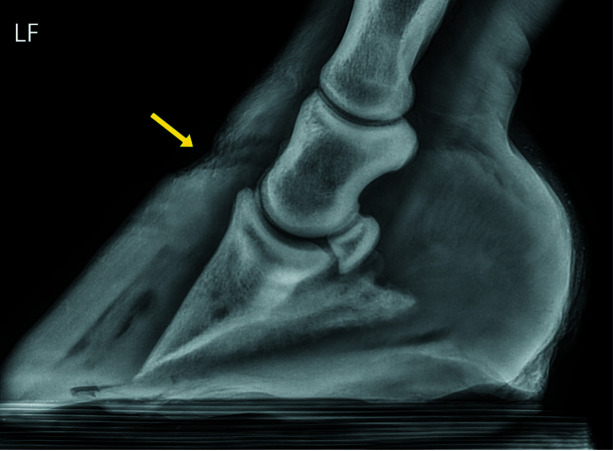
A radiograph of the left front foot (LF, hoof #13) obtained from a horse with severe laminitis, prior to euthanasia, with evidence of sinking of the pedal bone (yellow arrows) within the hoof capsule. Radiographs were taken by Ballarat Equine Clinic and are provided here with their permission.

**Figure 14:**
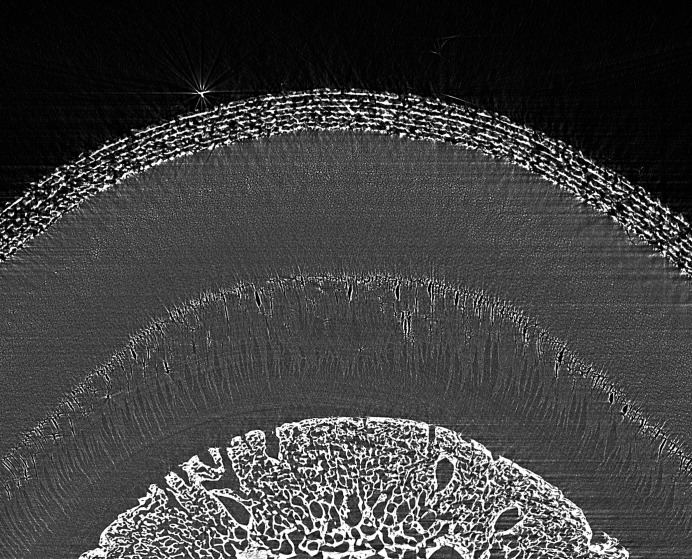
Hoof #13 of the laminitis horse, zoomed view with no contrast. This hoof (left front) is from the same horse as in Figs. 11 ▸ and 12 ▸. The lamellae are no longer parallel. Some of the lamellae are shortened and others are thickened and distorted. The darker areas may represent areas of serum accumulation.

**Table 1:** Source (AB = abattoir; BE = Ballarat Equine), preparation and data collected from 13 equine cadaver feet All 13 feet were imaged with cCT. Hooves #12 and #13 were from the same horse, which was euthanized because of severe laminitis.

Hoof #	Source	Fresh/frozen	Front/hind foot	Perfused	Type of contrast	Synchrotron CT
1	AB	Fresh	Front	Yes	Visipaque	Yes
2	AB	Fresh	Hind	Yes	BriteVu	No
3	AB	Fresh	Front	Yes	BriteVu	Yes
4	AB	Fresh	Front	Yes	BriteVu	No
5	AB	Fresh	Hind	Yes	BriteVu	Yes
6	AB	Fresh	Hind	Yes	BriteVu	No
7	AB	Fresh	Front	Yes	BriteVu	No
8	AB	Fresh	Hind	Yes	BriteVu	No
9	AB	Fresh	Front	No	N/A	Yes
10	AB	Frozen	Front	No	N/A	Yes
11	BE	Frozen	Front	No	N/A	No
12	BE	Frozen	Front	No	N/A	Yes
13	BE	Frozen	Front	No	N/A	Yes

**Table 2:** Settings of sCT scans of hoof #9 acquired at the Australian Synchrotron IMBL to determine optimal energy and distance from the detector for subsequent scans The area of interest was the equine lamina, which connects the hoof bone to the hoof capsule.

Scan #	Energy (keV)	Pixel size (µm)	Detector distance (m)
1	80	12	6
2	60	12	6
3	70	12	6
4	80	12	6
5	55	12	6
6	55	12	3

**Table 3:** Specimen characteristics and the type of sCT scans acquired of equine cadaver feet at the Australian Synchrotron IMBL All scans were performed with 55 keV and 6 m distance from the detector. Short scans were one slice (one full specimen rotation) through the hoof. Long scans were scans of the entire hoof from the sole to the coronary band.

Hoof #	Fresh/frozen	Contrast	Short scan	Long scan	Laminitis
1	Fresh	Visipaque	Yes	No	No
3	Fresh	BriteVu	Yes	No	No
5	Fresh	BriteVu	Yes	Yes	No
9	Fresh	None	Yes	No	No
10	Frozen	None	Yes	No	No
12	Frozen	None	Yes	No	Yes
13	Frozen	None	Yes	Yes	Yes
